# A History of Medical Liability: From Ancient Times to Today

**DOI:** 10.7759/cureus.41593

**Published:** 2023-07-09

**Authors:** Petroula Mandilara, Spyridon P Galanakos, George Bablekos

**Affiliations:** 1 Attorney at Law, Athens Bar Association, Athens, GRC; 2 Department of Orthopaedics, Primary Health Care Corporation, Athens, GRC; 3 Department of Biomedical Sciences, Occupational Therapy and Nursing, University of West Attica, Athens, GRC

**Keywords:** medical liability, liability, responsibility, medical practice, lege artis, medical act

## Abstract

Medical liability is a term associated with medical procedures and acts that has been a topic of controversy and research since ancient times all over the world. In ancient times, it was usually explained based on theocratic conceptions and was directly related to the social position of the patient to whom the physician applied medicine, while the capacity of the physician implied specific qualities. The present review of ancient history and geography provides a detailed description of the issues concerning medical responsibility through the years in ancient Greece, the birthplace of Asclepius and Hippocrates, the fathers of medical science, and the medical oath of conscientious performance of physician duties. Furthermore, it discusses the issue of medical liability globally from ancient times till more recently. Finally, medical liability and the interaction of its various aspects in Greece in recent years is presented according to Greek legislation to provide a good review for the reader to acknowledge the changes in medical liability through the centuries.

## Introduction and background

Medical liability is not a modern concern but has been a topic of thought and controversy for humanity throughout world history [1]. A brief historical overview of medical responsibility can contribute to a better understanding of the phenomenon as well as the perception of important parameters of modern reality. Historically, the correct way of practicing medicine was not always certain, and only when these frameworks were delimited did the practice of medical science become clear in accordance with the rules. Moreover, only when this step was taken, could progress be made in the field of medical responsibility as the requirement for respect and medical liability as the result of medical malpractice. Before this, different opinions were expressed about the irresponsibility of doctors or the limitations of their responsibility. Throughout centuries, civil liability, being connected to the medical profession, goes along with the cultural index of the time [2].

Furthermore, the final characterization of an operation as a failure and the possible legitimacy of each medical operation that affects the subsequent liability of the physician are not related to a single criterion but are a result of several factors. Certainly, the ultimate responsibility of the physician is not based on deviation from one predetermined pattern of behavior but is judged a priori and according to the result. The reference to the distinctions of medical acts is related to the issue of their legal characterization as some of them are finally allowed while others are not [1].

In modern times, perceptions have changed and doctors are now responsible for their actions and omissions, a responsibility that in essence protects the very value of medical science as a component of human culture and its practice as a profession because it contributes to showing the maximum possible diligence and attention in the medical profession and function.

The aim of this narrative review was to historically illustrate the issue of civil liability, along with its induced consequences, as a result of applied medical management throughout ancient times. It should be pointed out that during Greek antiquity, the matter of medical liability was completely unknown and under no circumstances related to the current healthcare dilemmas and controversies, from both legal and medical scientific documentation.

## Review

Historical overview

In 2400 BC, medical science was flourishing in the countries of Mesopotamia. Babylon became the center of medical development. The first legislative provisions for the practice of the medical profession, that is, the kind of care that the physician should show and the structure of the doctor-patient relationship, are contained in texts thousands of years before Christ and specifically in the Babylonian Code of Hammurabi (1900 BC), the “Venice,” the sixth book of Asvesta of the ancient Persians, and the “Holy Bible” of the ancient Egyptians. For example, in the texts, it is stated that if the doctor injured his eye and lost his sight, he would be punished by amputation of his hands. The Hammurabi Code also mentioned particularly severe penalties for a doctor who would inadvertently injure a patient during surgery. Specifically, of the 250 articles of the Code of Hammurabi, 20 have an indirect or a direct reference to doctors, as well as to medical fees (articles 206-208), abortions (articles 209-214), serious surgeries (articles 215 et seq.), and other medical matters of the time [3].

It is worth mentioning that in the Code of Hammurabi, the basic guideline of the doctor’s debt is outlined, which is the provision of benefit to the patient and the principle of equal compensation. In other words, if the doctor treats the patient, he should be rewarded, while if he is to blame for injury or death, he should be punished. More specifically, articles 218 et seq. of the above Code state that “If a doctor has performed a serious operation with the bronze scalpel on the body of a nobleman and saved his life or if he has operated on the eye of a gentleman with the scalpel and has saved him the eye, then receive as a reward 10 silver shekels ... If it is, on the contrary, a common citizen, the doctor’s fee to be 5 shekels. If it is a slave, the master of the slave is to pay the doctor 2 silver shekels … If a doctor has performed a serious operation with the bronze scalpel on the body of a nobleman and has caused his death or has opened an abscess in the eye of a nobleman and this has caused the loss of the eye, to cut off his hand…” [3]. Therefore, it is obvious that the basic principle of penal law in the Hammurabi Code was the principle of *lex talionis*. The symbolic retribution of equals to any offender is found in most crimes of the Code. Moreover, although the Code places great emphasis on intentional criminal acts, the provisions relating to medical interventions are severely punished as acts committed through negligence [1,2].

Furthermore, references to medical liability can also be found in ancient Egyptian texts. According to the provisions of the Holy Bible, the doctor undertook the treatment of the patient without any responsibility, at the risk of the patient for the first three days, while after three days, the treatment continued, but the doctor had the same responsibility. The provision of the Holy Bible of the Egyptians is worth mentioning, where they treated the matter of medical negligence with an extremely modern look for that time, as the doctor was punished with the death penalty if he did not follow the rules of his science, regardless of outcome, surgery, or treatment. The lege artis treatment was favored overall and would determine the discharge or conviction of the therapist. Moreover, it should also be mentioned that two Greek authors, Diodorus Sicilus and Aristotle, described medical liability during the Pharaohs’ reign. Specifically, provided that patients’ management followed necessarily the rules being reported in texts kept in places of worship, doctors would not be punished even in the failure of the applied medical treatment, which would not happen in case the aforementioned rules were not taken into account even for patient’s benefit [2,3].

Regarding the ancient Greeks, which will be analyzed later, the following should be noted: even though they contributed decisively to the progress of medical science and were pioneers for their time in this field, there are no specific reports on medical responsibility. Conclusions about the responsibility of doctors can be drawn from other sources, which seem to refer to the irresponsibility of doctors [4].

In Rome, the problem of medical liability arose early and over time received different solutions. More specifically, although Pandects, where there is a list of possible medical errors, states that the failure of treatment could never be considered a reason for medical liability, the Roman laws of the Dodecanese era stated that physicians should be punished for their negligence and inexperience. It is worth noting that as early as the year 573 from the founding of Rome, the conditions for the evidence of the doctor’s liability for the patient’s compensation had been formulated. Of note, the conventional responsibility of the doctor does not seem to exist in Rome [1,2].

In the Roman empire, physicians, many of whom were Greek living under conditions of slavery, apart from diagnosis and treatment, also knew how to brew suicide potions, something which happened often at the time, thus dragging the application of the Lex Romana. Moreover, physicians were frequently involved in murders related to hereditary issues, being, according to Pandects, the most usual suspects and/or accomplices. Besides, in case of an unintentional medical error, the applicable Roman law did not punish negligent damage from the physician’s side, even if there was death, but it was liable to compensation for damages “a sich” [5].

The Roman years also show important laws, such as those of the “Dodecadel” (449 BC), “Lex Aquilia Plebiscitum” (180 BC), “Lex Cornelia de Sicariis et Veneficiis,” Lex Pompeia de Parricidiis,” and “Lex Julia de vi Publica et Private,” which are dated around the 1st century BC. The common denominator of all the above is the first attempt to define the civil and criminal responsibility of the doctor, even at an early stage, as well as the realization of the inability to prove the medical error. Roman laws set their own rules for physicians. For doctors coming from the upper social classes, negligence or incompetence was punished with exile, for doctors coming from the lower social classes, negligence and incompetence were punished with death [6].

Moreover, the Justinian Code, which appeared in Rome between 529 and 564 AD, included within its provisions a precept that indicated that a medical expert would not be used to the proper or greatest advantage if he were to be simply regarded as an ordinary witness, appearing for one side or the other. The Code, with much wisdom for the time, stated that the function of such an expert was really to assist the judiciary by impartial interpretation and opinion based on his specialized knowledge [2,3].

The Greco-Roman years were marked by two especially important personalities, the physician and philosopher Galen (129-199 AD) and Soranos the Ephesian (first half of the 2nd century AD). The first of these combined in his written texts his scientific references both to purely medical issues and issues of medical ethics. The relevant system based on his writing was called “Galinism.” On the other hand, Soranos the Ephesian was particularly concerned about surgery and pharmacology. He was considered the father of gynecology with a significant contribution to obstetrics and embryology. His work on “female passions” is fundamental and influenced medical science until the 16th century through Latin, German, French, English, Dutch, and Spanish translations or adaptations [1,2].

Furthermore, according to the law of the Visigoths and the Ostrogoths (5th to 6th century AD) as well as the law of the Crusaders, the position of physicians can be considered particularly precarious, as in the first case, physicians were entitled to claim remuneration only if the medical procedure they had performed was successful, while in the case of failure, their medical responsibility arose. In the case of failure, the doctors were responsible even if they had not faithfully followed the wrong instructions given by the patient [2,4].

In Byzantium, where the Church played a dominant role and theocratic views continued to prevail, arose the belief that the saints protected faithful people from demons, magic, and diseases. The main features of Byzantine medicine were the concept of closed nursing care with the establishment of the first hospitals and the implementation of social welfare measures, mainly with the establishment of auspicious institutions (nursing homes, orphanages, orphanages). Byzantine law regulated the criminal liability of doctors, the punishment for whom was a function of the existence of deceit, the intensity of negligence, and even the social position of doctors and patients. The existence of a medical error was proven only when expert research was carried out, with several penalties pointed out for the doctors, such as a fine, confiscation of his property, deportation, and, rarely, even death by a sword [7].

From the 11th to the 15th century, medicine was practiced uncontrollably, while after the French Revolution (1789), medical liability was treated on a purely legal basis, taking the formality of law. Moreover, the founder of the Forensic Medicine Society in Paris, Legrand de Sauce, in 1874, declared: “No one has the right to be above the law. And the doctor is confident enough that he will not seek an exception to this.” During the years after the 15th century AD, the perception of irresponsible physicians prevailed, especially in the interest of science and progress [1,2].

In Western Europe, during the Middle Ages, failed medical treatments resulted in the use of the death penalty. On the contrary, from the 12th AD until the 17th century AD, the irresponsibility of the doctors applied, as the patient was responsible for any medical errors due to poor choice of his treating physician. From the 18th century AD, the complete impunity of doctors ceased and criminal responsibility for medical errors made its appearance, which included imprisonment sentences, as well as the ancillary penalty of prohibition to practice the profession [2].

An undoubtedly major historical development in medical liability was the establishment of the Nuremberg Code. Following the Nuremberg Trials in 1945-1946, in which it was decided to condemn the doctors of the Nazi regime for inhumane experiments on prisoners, the above-mentioned code was created, which for the first time provided for medical procedures to be performed only after obtaining consent, that is, it established the principle of autonomy. This was followed in 1964 by the proclamation of the World Health Organization in Helsinki and in 1987 by the European Code of Medical Ethics. We should not forget the European Convention on Human Rights in Biomedicine, known as the “Oviedo Convention,” which was ratified by Law 2619/1998, has supra-legislative power and provides for medical liability in art [8].

Regarding this brief historical overview, it is obvious that the rules and principles that had to be followed for the proper practice of medical science and practice were not originally prescribed. The wrong practice of medicine was not always linked to legal liability and of in each case the obligations of doctors were not determined in advance. A prerequisite for progress in the field of legal liability was the development of the positive and medical sciences and the acquisition of necessary knowledge for the function of the human body. As medical liability follows medical practice, it was necessary to first determine the rules of practice of medical science and practice (lege artis) and consequently to determine what is done within the appropriate framework and in accordance with the relevant rules. Only when the scientific foundations of medical research and practice were established in recent centuries, it broke free from possible religious superstitions. At that time, the legal liability of doctors became the subject of substantial theoretical, legislative, and jurisprudential processes.

Medical liability specifically in ancient Greece

Regarding ancient Greece, more specifically, it should be mentioned that medicine is inseparably linked to the presence of man on earth due to the need for wound healing and disease therapy [1,2,4]. It is known that only a few Greeks pass down important manuscripts concerning contemporary medical liability. The most known laws of that period were written by Solon and Lycurgus which got lost through the centuries. Therefore, the only available text, pertaining to the city of Corinth, does not refer explicitly to medical liability. The famous philosophers of ancient times, Plato and Aristotle, expressed entirely opposite views on the issue of medical liability. Plato supported that if the doctor did his best to manage the disease and prevent a patient’s death, that alone acquits him and frees him from every possible accusation. Aristotle, on the other hand, formulated that for a doctor the adequacy of medical knowledge and the effectiveness of the applied curative treatment can be both judged exclusively by another doctor. The latter made Aristotle to be considered the founder of the current Western judging system concerning medical liability. Furthermore, according to Aristotle, when managing a patient, the specificity of each case should be taken into account to select the appropriate medical treatment by avoiding the standard application of the rules reported in medical books, which is the Egyptian way of acting. However, it should be pointed out that Aristotle examined the issue of medical liability from a philosophical rather than a legal approach [9].

The way medical liability was addressed in ancient Greece can be fully understood by the following example found in ancient texts. Arrian - Flavius (historical writer, philosopher, and political and military person), born and died in the city of Nicomedia (Bithynia, Asia Minor) about 95 AD and 175 AD, respectively, reported the execution by crucifixion of a physician named Glaucos in the Court of Alexander the Great due to prescription of a wrong medication to a mad man named Hephaestus who was a beloved friend of Alexander the Great. Nevertheless, according to Plutarch (46-12 BC), Hephaestus’s death should not be rendered to Glaucos by himself, but in Hephaestus’s disobedience to accurately follow his medical advice. Moreover, the decisions of Alexander the Great on focusing on Glaucos’ crucifixion as well as on the destruction of the temple of Asclepius along with the fire of the city of Persepolis to honor his dead friend Hephaestus were false and subjective judgments [1,2].

In addition, in ancient Greece, until the 8th century BC, medicine was inextricably linked with religion. Therefore, if the doctor, at that time, deemed the patient’s illness as incurable, he referred him to the Asclepieia to priest doctors, resulting in the irresponsibility of the doctors. In ancient times, medical art was practiced in special temples, named Asclepieia, by special priests named Asclepiads. Αsclepiads were of aristocratic descent related immediately to Asclepius, either directly in the natural way or indirectly in a spiritual way through the teaching of Asclepius. Additionally, it should be pointed out that, in ancient times, Apollo and Asclepius were both considered spiritual mentors of Asclepiads [10].

It should be mentioned that the first medical institutions and centers of medical knowledge in ancient Greece were the Asclepieia, the first medical institutions in history that inaugurated the art of Asclepius. In ancient times, the function of the Αsclepieia was defined by a vague boundary between superstition and science. The latter is proved by archeological data from the Asclepieia themselves, such as texts, stone offerings, and inscriptions. This is crucial proof of the combined divine and human knowledge used in the treatment of patients managed by the Asclepiads. In addition, the beneficial effects of the climate and the sun, particularly for infectious diseases, were widely known, from Asclepius and Asclepiads, being included in the curative treatment of such a patient. The health of the human being reflects the balance established between the body (tangibility) and the spirit (soul), given that the disorder of the aforementioned balance leads to illness, according to either Asclepius or Hippocratic and French medicine [11].

In Asclepieia, the cornerstone for a successful cure was the combination of a patient’s faith in the effectiveness of the applied curative treatments, along with the conviction of their divine origin. The medical practice, in Asclepieia, was based on the administration of medicaments, including poisons with their antidotes, as well as ointments prepared from plant roots having healing properties, associated with surgical interventions. According to the evidence originating from temples, “surgeon” meant the use of hands to achieve curative treatment for various ailments of the human body. Moreover, either the four humors theory of Hippocratic medicine (phlegm, blood, yellow bile, and black bile) or the four elements theory of the pre-authoritarian philosophers (air, earth, water, and fire) were found in the Asclepieia. The latter, that is, the four elements theory, supports that raw materials of nature take part in the construction of the human body. As a result, disorders in bonds connecting the aforementioned materials lead to disease emergence. In addition, the term physiology seems to determine and explain the interactions between the healthy human body, on the one hand, and nature, on the other [12].

Furthermore, according to the Asclepiads’ medical thought, the selection of the applied curative treatment depended on the observation of the clinical symptoms, thus contributing to the understanding of the pathogenesis of the emerging disease. Moreover, there are no findings for payment regarding the Asclepiads’ provided services, except for the pledge to God to serve human life, which confirms and enhances the humanistic role of medical science in ancient times. To be considered worthy to become an Asclepiad, despite their status as priests, Asclepiads had to own virtues such as faith, justice, temperance, modesty, and freedom and they thought neither there was a competition between religion and science, nor did they confuse knowledge with magic or any fictional elements. A basic element of the Asclepiads mentality and education was the critical thinking used to approach and resolve any functional problem of human nature, which requires medical intervention. The latter supports the concept that, throughout Greek antiquity, the meaning of the miracle, although widely believed by the simple people, nevertheless, according to Asclepiads’ side, it did not exist while efforts were made to explain any questions with inductive thinking and medical knowledge [13].

A century later, the Dragon legislator enacted laws that provided for the liability of physicians with severe sanctions, such as corporal punishment and the death penalty, while in the 6th century BC, Solon enacted new laws that moved away from the hitherto prevailing theocratic conception. A milestone in the history of medicine was in the 5th century BC when Hippocrates, as the founder of medical ethics, systematized diseases and their treatment methods and distinguished medicine from prejudice and demonology [14].

In ancient Greece, the main task of Hippocratic physicians was to heal the appeared disease, rather as craftsmen than as expert scientists in today’s sense of the term. Moreover, the subsistence needs of Hippocratic physicians were covered by the treatment of their patients either in the private space of the physician or in different places, as physicians usually traveled searching for patients, which had nothing to do with the current reality, scientific and professional, of a contemporary physician. Regarding the physicians’ social status, in Greek antiquity, they were treated as any other craftsmen as there were neither exact scientific qualifications determining the required knowledge for becoming a doctor, nor certifications concerning medical education along with the acquired skills and/or experience with the passage of time. In addition, in ancient times, there was no legislation for liability and malpractice in the medical profession, while physicians were differentiated from other craftsmen exclusively by their reputation [4]. Nowadays, the issue of malpractice could be defined as a professional error that produces a prejudice to the patient, which may trigger civil, administrative, and criminal liability.

Furthermore, issues such as medical malpractice and/or medical negligence, wherever mentioned in the Hippocratic texts, seem to be of a rather historical and ethical value, especially if an attempt is made to compare them with the current jurisprudence focusing on medical negligence. Moreover, Hippocratic texts are written in the form of instructions, presenting the proper intervention that the physician must apply to cope with the disease. According to Hippocratic texts, the possibility of an error on the part of the physicians was very frequent, thus as great doctors were nominated only those performing the fewest medical mistakes. Moreover, it is interesting to report the role of the disease by itself, concerning the characterization of a medical fault. Specifically, according to the “Affections” script, the acuteness and the severity of a disease, both strongly contributing to the weakening of the human body, are blamed for a negative clinical prognosis rather than a physician’s fault. A physician’s responsibility, at that time, was recognized on the basis that the patient was treated incorrectly or out of ignorance being defeated by the disease [2,15,16].

In the Hippocratic texts, except for the references to issues such as medical practice and/or medical negligence, references to advocacy for physicians are also found. It is well known that unpredictable complications, associated with adverse events, may occur during the medical management of many diseases, without this constituting a physician’s fault. In this matter, the opinion reported in the Hippocratic texts is that the physician is not able to determine and control everything about healing. Besides, an interesting point of view in the texts is that although physicians are usually accused of any negative event that emerged during curative treatment, they also lack the praise of patients in case they improve. The latter is attributed to the conviction that the improvement of health depends on the natural course of the disease by itself, and is not related to a responsible physician’s medical intervention [17].

Moreover, in ancient Greece, relevant references to medical liability and medical errors can be found in the legislative texts of the Dragon (621 BC) and Solon (639-559 BC). These texts mitigate the cruelty of the former ones who imposed severe punishments, even the death penalty for physicians, and introduced a less theocratic view of medical science. Throughout time and universally, the contribution of Hippocrates (460-377 BC), during the ancient Greek years, would mark the history of medical science, and his texts, which were progressive for their time, would influence modern theory and practice. Studying the famous “Oath of Hippocrates,” it can be stated that the issues of medical ethics and bioethics are already raised, such as the prohibition of abortion and euthanasia and the obligation to aid patients [18].

Aspects of medical liability

Medical liability relates to cases in which an undesirable medical result is due to a violation of fundamental recognized rules of medical science in combination with actions (acts or omissions) contrary to the objectively imposed duty of care. Thus, medical liability arises when “the doctor did not pay the objectively obliged care and diligence, which any moderately prudent and conscientious person could and should have paid under the same real circumstances, based on legal rules, conditions prevailing in transactions and in the ordinary course of business experience and logic and at the same time there is a causal link between the medical act or omission and the criminal unintended result.” In the past, attempts to conceptualize medical liability have led to the formulation of theories of “fully irresponsible,” “sui generis reduced liability,” and “full liability of the physician” [19].

The first theory of the exclusion of medical liability appeared in the early 19th century, and according to it, physicians are not legally responsible for mistakes they make in the performance of their duties unless there is a fraudulent act. Proponents of this theory have been working to make the actual transcript of this statement available online. The second theory spoke of the introduction of reduced liability of the physician only when there was deceit or gross negligence and not a slight one. The privileged status that this theory provided to physicians led to its abandonment. The prevailing view today is in favor of the third theory, according to which the doctor is subject to the rules of common law. Indicatively, the Administrative Supreme Court of France, in its decision in 1862, decided that “every person, regardless of his position or profession is subject to this rule and that the doctor is no exception.” Consequently, the acts or omissions committed by the doctor are submitted to the courts, where they are judged in accordance with the applicable legal provisions [20].

The action of doctors today is not uncontrollable but is subject to certain conditions of legal liability. At times, different views have been held regarding the responsibility of physicians. One of these views is in favor of irresponsible doctors. According to this position, doctors cannot be subject to scrutiny before authorities and courts because the existence of such responsibility prevents them from exercising their function and ultimately hinders the progress of science and effective treatment and care of the patients. However, this position has been criticized because it is opposite to the basic requirements of the law, as it is extremely difficult or impossible to establish irresponsibility for a category of professional activity or function. Another view wants doctors to be held responsible for medical acts (or omissions) that take place while performing their duties, with the result that the provisions of common law apply to them, while the latter shows doctors to be responsible only for gross negligence [21].

Categories of Medical Liability

The legal liability of physicians for acts or omissions that occur during the performance of their duties is categorized into civil, penal, and disciplinary [22]. This classic distinction is causally related to the field of law that regulates the legal consequences of their liability. Therefore, civil liability is referred mainly to the doctor’s liability for compensation when the doctor during the exercise of his duty caused some damage to a patient. Criminal liability is related to the respective criminal sanctions that result from the acts or omissions of the doctor, while disciplinary liability has to do with the public (excluding criminal) law consequences related to the disciplinary offenses imposed on the doctor by disciplinary bodies [22].

The Interaction of Different Forms of Legal Liability With Each Other

Even though penal medical liability had preceded and piqued the interest of legal theorists, law enforcement and legal practitioners, holding the forefront of medical liability in the Greek legal system, civil medical liability has begun to monopolize the area of ​​responsibility of doctors. Concerning the relationship between civil and penal liability, it is crucial to note that these are two distinct concepts with several differences between them, as differences are identified in the sources of each form of liability, the purpose they serve, the degree of guilt, the consequences provided for [23].

Penal liability derives from the rules of criminal law which are characterized as public policy, while civil liability is derived from the provisions of civil law and civil and private law in general [22,23]. Furthermore, the purpose of penal liability is either to prevent or suppress the commission of illegal acts through the imposition of a penalty on the perpetrator. On the contrary, the nature of civil liability is restorative, as it intends to restore the injured party (in this case the patient) to the state where would have been if the damaging event had not taken place (i.e., the medical act). Regarding the degree of responsibility, the following should be noted: penal liability necessarily presupposes the existence of fault (deceit or negligence), while civil liability can also be evidenced in cases of objective liability, liability at risk, and liability for liability of third parties (alien acts). The penal penalties imposed, which are of a personal nature (and therefore not hereditary) are either pecuniary or deprivation of liberty, while the compensation, the consequence of civil liability, is of a property nature and is inherited. Another point that can be considered to link in some way civil liability with penal liability is the representation of civil action, an institution that has its roots in civil law and has been introduced in criminal proceedings to strengthen the position of the victim or other actively legitimized persons [24,25].

Disciplinary liability has different conditions (breaches of duties and obligations imposed on physicians by the Code of Medical Ethics) and purposes (ensuring the validity of the medical profession) and leads to different sanctions compared to both civil and criminal liability. In fact, it is the responsibility of the doctor toward professional organizations, for example, the medical associations of the country. The purpose, however, of this paper is not to delve into further issues of this liability.

In any case, once all legal conditions are met, it is possible that civil, penal, and disciplinary liability may arise at the same time for the same facts, with the result that even if a criminal prosecution or lawsuit has arisen in the civil courts, this does not suspend or prevent disciplinary action and vice versa.

## Conclusions

One term that is at the heart of medical liability is “failed medical practice” which is referred to as a “medical act,” which refers to a (positive) act or omission of the doctor regarding a specific person/recipient of his services. The characterization of the medical act as “failed” is attributed to the medical act when it results in harm to the patient, that is, the infringement of his legal rights (life, physical integrity). It is crucial, therefore, for the acquisition of medical liability to distinguish the damage that is actually related to the act or omission of the doctor from the damage that results from the inevitable development of the patient’s health, despite given doctor’s care.
